# Cost-effectiveness and cost–benefit analyses of fluoride varnish for caries prevention in Guangxi, China

**DOI:** 10.1186/s12903-024-04220-x

**Published:** 2024-05-09

**Authors:** Liying Tang, Shengjie Nong, Kun Chen, Qiulin Liu, Xueting Yu, Xiaojuan Zeng

**Affiliations:** 1 College of Stomatology, Hospital of Stomatology, Guangxi Medical University, Nanning, Guangxi China; 2 Department of Oral Health Policy Research, Guangxi Medical University, Nanning, Guangxi China

**Keywords:** Dental caries, Fluoride varnish, Decision support models, Cost–benefit analysis, Cost-effectiveness analysis, Prevention and control

## Abstract

**Objectives:**

The objectives of this study were to evaluate the cost-effectiveness and cost-benefit of fluoride varnish (FV) interventions for preventing caries in the first permanent molars (FPMs) among children in rural areas in Guangxi, China.

**Methods:**

This study constituted a secondary analysis of data from a randomised controlled trial, analysed from a social perspective. A total of 1,335 children aged 6–8 years in remote rural areas of Guangxi were enrolled in this three-year follow-up controlled study. Children in the experimental group (EG) and the control group (CG) received oral health education and were provided with a toothbrush and toothpaste once every six months. Additionally, FV was applied in the EG. A decision tree model was developed, and single-factor and probabilistic sensitivity analyses were conducted.

**Results:**

After three years of intervention, the prevalence of caries in the EG was 50.85%, with an average decayed, missing, and filled teeth (DMFT) index score of 1.12, and that in the CG was 59.04%, with a DMFT index score of 1.36. The total cost of caries intervention and postcaries treatment was 42,719.55 USD for the EG and 46,622.13 USD for the CG. The incremental cost-effectiveness ratio (ICER) of the EG was 25.36 USD per caries prevented, and the cost–benefit ratio (CBR) was 1.74 USD benefits per 1 USD cost. The results of the sensitivity analyses showed that the increase in the average DMFT index score was the largest variable affecting the ICER and CBR.

**Conclusions:**

Compared to oral health education alone, a comprehensive intervention combining FV application with oral health education is more cost-effective and beneficial for preventing caries in the FPMs of children living in economically disadvantaged rural areas. These findings could provide a basis for policy-making and clinical choices to improve children’s oral health.

## Introduction

The burden of dental caries has long been a significant global public health challenge [1]. In 2010, untreated caries in permanent teeth were identified as the most prevalent disease worldwide, affecting 2.4 billion individuals [2]. The cost of treating dental caries has imposed a substantial economic burden on both families and health care systems [3]. Moreover, untreated caries can compromise not only mastication but also speech, smiling, and psychosocial well-being, significantly impacting the quality of life of children and their families [4]. Despite decades of research and public health initiatives aimed at reducing the prevalence, dental caries remains widespread [1]. This highlights the need for a public health approach that emphasises effective, cost-efficient, safe, and widely accessible interventions to address this pervasive issue.

Newly erupted first permanent molars (FPMs) are more susceptible to caries due to their lower level of enamel calcification, the complexity of the oral environment, and the challenge of promptly removing food debris [5]. The essential role of FPMs in absorbing occlusal forces and forming the foundation of adult dentition has led to increasing concerns about the high incidence of caries in these teeth. This situation is further aggravated in rural areas, where economic challenges and limited access to dental care further compromise oral health outcomes [3, 6]. A survey of health institutions in Guangxi revealed a continued shortage of dental human resources, with many township hospitals lacking dental departments or specialists [7]. In many rural parts of Guangxi, which are characterised by delayed economic development and significant workforce migration to urban centres, there is a high number of left-behind children who are typically cared for by grandparents or relatives. Due to the lack of parental supervision and guidance, this demographic is particularly vulnerable to dental caries, underscoring the importance of preventing and controlling this condition [8].

Both pit and fissure sealants and fluoride varnish (FV) have been shown to be effective in preventing caries in FPMs [9]. However, pit and fissure sealing requires professional dental equipment and a high level of operational precision, making it less accessible in resource-constrained settings. In contrast, FV, with its straightforward application and minimal requirement for professional dental equipment, may be more suitable for environments with limited resources [10]. School-based oral health education interventions can help enhance children’s knowledge of oral health, yet their effectiveness in reducing the incidence of dental caries is not sufficiently robust [11, 12]. The application of FV, in addition to oral health education, has emerged as a promising strategy for preventing dental caries in children. This combination is theorised not only to provide a direct therapeutic benefit through the application of fluoride but also to empower children and their guardians with the knowledge needed to maintain good oral hygiene practices [12, 13]. However, the economic justification for integrating such interventions into public health programmes, especially in rural and economically disadvantaged regions, remains underexplored.

Given the scarcity of health care resources, decision-makers must utilise health economics to identify the most cost-effective prevention and treatment programmes [14]. Despite the recognised need, economic evaluations remain underutilised in dentistry [14]. Cost-effectiveness analysis (CEA) and cost-benefit analysis (CBA) are pivotal tools for assessing the economic value of medical interventions and public health projects, playing an increasingly vital role in optimising resource allocation and enhancing the efficiency of health interventions. As the field of health economics continues to evolve, these analytical methods will provide more scientific and precise support for the formulation of global health policies and the management of health resources [15]. Therefore, we have incorporated CEA and CBA into our study. The present study aimed to address this gap by performing a thorough CEA and CBA of oral health education combined with FV application versus oral health education alone to enable the judicious allocation of limited public health resources and improvements in oral health outcomes for vulnerable populations to a maximal extent.

## Methodology

This study adhered to the Consolidated Health Economic Evaluation Reporting Standards (CHEERS) guidelines. CEA and CBA were the forms of economic evaluation employed to compare two preventive options for managing FPM caries: one incorporating FV application plus oral health education and the other comprising oral health education only. This study was a secondary analysis of data from a randomised controlled trial that received ethical approval from the Ethical Review Committee of the Chinese Stomatological Association [8]. The analysis in this study was conducted from a societal perspective.

### Sample size and participants

Calculations were performed using PASS software version 11.0 (NCSS, LLC, Kaysville, UT, USA), with an anticipated caries prevalence of 9% in the experimental group (EG) and 15% in the control group (CG). With a significance level (α) of 0.05 and a power (1-β) of 90%, the minimum required sample size for each group was 615 children. Accounting for an expected dropout rate of 15% during the study period, the total required sample size for each group was adjusted to 708.

Simple random sampling was utilized to select 9 schools from 325 public schools in Dahua County, Guangxi, China, encompassing a total of 32 classes. Children from these classes who met the eligibility criteria were invited to participate in the study. The inclusion criteria were healthy children aged 6–8 years with a decayed, missing, and filled teeth (DMFT) score of 0, no acute or chronic systemic diseases, no gingivitis or ulcers, no history of asthma, and no allergies who were not participating in other trials during the study period. Children with dental fluorosis or developmental enamel defects and children who had received pit and fissure sealants on their first permanent molars were excluded from the study. Participation required a signed parental consent form and the child’s written assent.

### Interventions

Children in the EG received oral health education twice yearly and were treated with 5% FV, while children in the CG only received oral health education twice per year. All interventions were conducted in school classrooms.

Oral health education was provided by the same dentist from the Hospital of Stomatology, Guangxi Medical University. Education included healthy eating and tooth brushing guidance; all children were advised to substitute candy and soft drinks with fresh fruits and vegetables. Additionally, the correct method of brushing teeth was demonstrated using dental models, and all children were encouraged to brush their teeth twice daily with fluoride toothpaste. Oral hygiene instructions were repeated every six months, resulting in a total of six sessions throughout the 36-month study. All children received free toothbrushes and toothpaste every six months, totalling six times throughout the study.

The children in the EG were scheduled to have FV applied at baseline and every six months thereafter, totalling six applications, which were conducted by two trained dentists from the Hospital of Stomatology at Guangxi Medical University. FV containing 5% sodium fluoride (Duraphat, Colgate-Palmolive (UK) Ltd., Waltrop, Germany) was used in this study. After brushing, the teeth were isolated with cotton rolls and dried with a cotton swab before the varnish was applied to all accessible surfaces of the FPMs using a disposable small brush. The remaining varnish was applied to other teeth in the mouth. The children were instructed not to drink or eat for half an hour, not to consume hard food for four hours, and not to brush their teeth on the day of application.

### Randomisation and blinding

Each child was assigned an ID number at the first visit, which was used for identification throughout the study. The ID numbers were recoded to generate a random number for each child; children with random numbers above the median were allocated to the EG, while those with random numbers below the median were assigned to the CG.

The personnel conducting the oral examinations and record-keeping were blinded to the allocation. Although informed about the allocation, the providers applying the varnish and their assistants did not participate in the oral examinations or record-keeping. Due to the distinctive physical properties of Duraphat, the participants were likely aware of their group allocation.

### Oral examinations

Oral examinations were conducted in schools at baseline and at the end of 36 months using a flat mirror and a CPI probe. All oral examinations and recordings were completed by two dentists from the Hospital of Stomatology, Guangxi Medical University. After brushing their teeth, the children lay supine while their tooth surfaces were dried using cotton rolls and swabs. The carious status of the FPMs was recorded according to the World Health Organization (WHO) standards [16]. The kappa values for both interexaminer consistency and intraexaminer consistency exceeded 0.80.

### Time horizon

The study was implemented from November 2014 to November 2017, spanning a total of 3 years.

### Currency, price date, and discount rate

In the analysis of historical cost data, all costs and effects were discounted at a 3% rate to adjust for inflation to 2017 Chinese Yuan (CNY) values and subsequently converted to 2017 US dollars (USD) using the November 2017 exchange rate of 1 USD = 6.62 CNY, as sourced from http://fx.sauder.ubc.ca/data.html.

### Health outcomes and Effectiveness

Our primary outcome measure was the caries increment (measured by the change in the DMFT index score) in the FPMs after 36 months of follow-up. The secondary outcome measure was the incremental prevalence of caries in the FPMs, measured as a percentage (%).

### Estimation of costs

From a societal perspective, this study included all costs, irrespective of the payer. The cost measurement encompassed both the intervention cost and the benefit measurement.

The intervention cost was calculated during the clinical trial process using the structural analysis method. Intervention costs were divided into two parts based on traceability: human resource costs and material costs. For the EG, the human resources included two trained dentists for the topical application of fluoride varnish, two dentists for dental examination and documentation, and one dentist for oral health education, allocating five minutes per child, three minutes per child, and 15 min per class (across a total of 16 classes), respectively. Additionally, two Centers for Disease Control (CDC) staff members were required to coordinate the work twice per year, dedicating approximately 378 min each time. The wage per minute for dentists and CDC staff was calculated by dividing the average annual wage of health industry staff, as reported in “The Guangxi Statistical Yearbook” from 2014 to 2017, by the total number of minutes worked in one year. The material costs for the EG included the cost of FV, toothbrushes and toothpaste, and examination instrument consumables. In the CG, the human resource cost and material cost of FV application were not included, with all other costs being the same as those in the EG.

Benefits were measured by calculating the average burden of caries disease. The disease burden of dental caries was divided into three components: direct medical costs, direct nonmedical costs, and indirect costs. (1) Direct medical costs were assessed as treatment costs at a tertiary teaching and research hospital in Guangxi, assuming cost homogeneity within China, as health technology pricing (for drugs, medical devices, and services) is regulated by the government. Direct medical costs included registration fees, imaging examination fees, filling costs, and material costs. (2) Direct nonmedical costs referred to additional expenses incurred by a child while undergoing treatment, such as transportation costs to and from appointments, assuming bus travel without transfers. (3) Indirect costs were related to productivity loss. As children require accompaniment by an adult for half a day to receive treatment, productivity loss was incurred by the adult, calculated based on the local average annual wage from “The Guangxi Statistical Yearbook 2017”.

### Cost-effectiveness and cost–benefit analysis

In CEA, the primary metric employed is the incremental cost-effectiveness ratio (ICER), which is the ratio of the difference in costs between two interventions to their difference in effects. The ICER is a valuable tool for assessing whether an intervention’s additional cost is justified by its effectiveness. In scenarios where an intervention is costlier yet more effective, an external benchmark, referred to as the willingness to pay (WTP), is utilised to determine cost-effectiveness. An intervention is deemed cost-effective only if the decision-makers’ WTP for the additional health benefits exceeds the intervention’s additional costs. Due to the absence of specific data on the WTP for preventing dental caries in children, this study approximated the WTP for caries prevention as equivalent to the cost of treating one averted caries incident.

CBA evaluates the economic outcomes of interventions by comparing the benefits (in terms of costs saved) to the costs incurred. The cost–benefit ratio (CBR) is the metric for this evaluation, representing the ratio of the intervention’s additional costs to the sum of the medical, transportation, and productivity loss costs that are averted.

This study used the number of caries as the outcome evaluation metric, with the ICER calculated as.

$$ICER = \frac{{{C_{EG}} - {C_{CG}}}}{{ - ({N_{EG}} - {N_{CG}})}}$$

The denominator is multiplied by -1 to convert the outcome into the number of caries prevented.

The CBR is calculated as.

$$CBR = \frac{{{C_{EG}} - {C_{CG}}}}{{N1(B1 + B2 + B3)}}$$

where.

C = intervention costs;

N = the number of caries;

N1 = the number of caries prevented;

B1 = the medical care costs averted per caries prevented;

B2 = the transportation costs averted per caries prevented;

B3 = the costs of lost productivity averted per caries prevented.

### Sensitivity analysis

Univariate and probabilistic sensitivity analyses were performed. To address the uncertainty in cost and benefit estimation, single-factor sensitivity analysis was used to evaluate the impact of changes in each parameter on the ICER and CBR. Except for the DMFT index score, the other parameters were changed within ± 20% of the base value, and a tornado plot was drawn. The Monte Carlo simulation method was used to simulate the parameters according to the triangular distribution 1,000 times, and probabilistic sensitivity analysis was performed to evaluate the effect of random values of each parameter on the ICER. TreeAge Pro 2022 software was used to establish a decision tree model and analyse the data.

## Results

### Baseline situation and caries prevention effect analysis

**Fig. 1:**
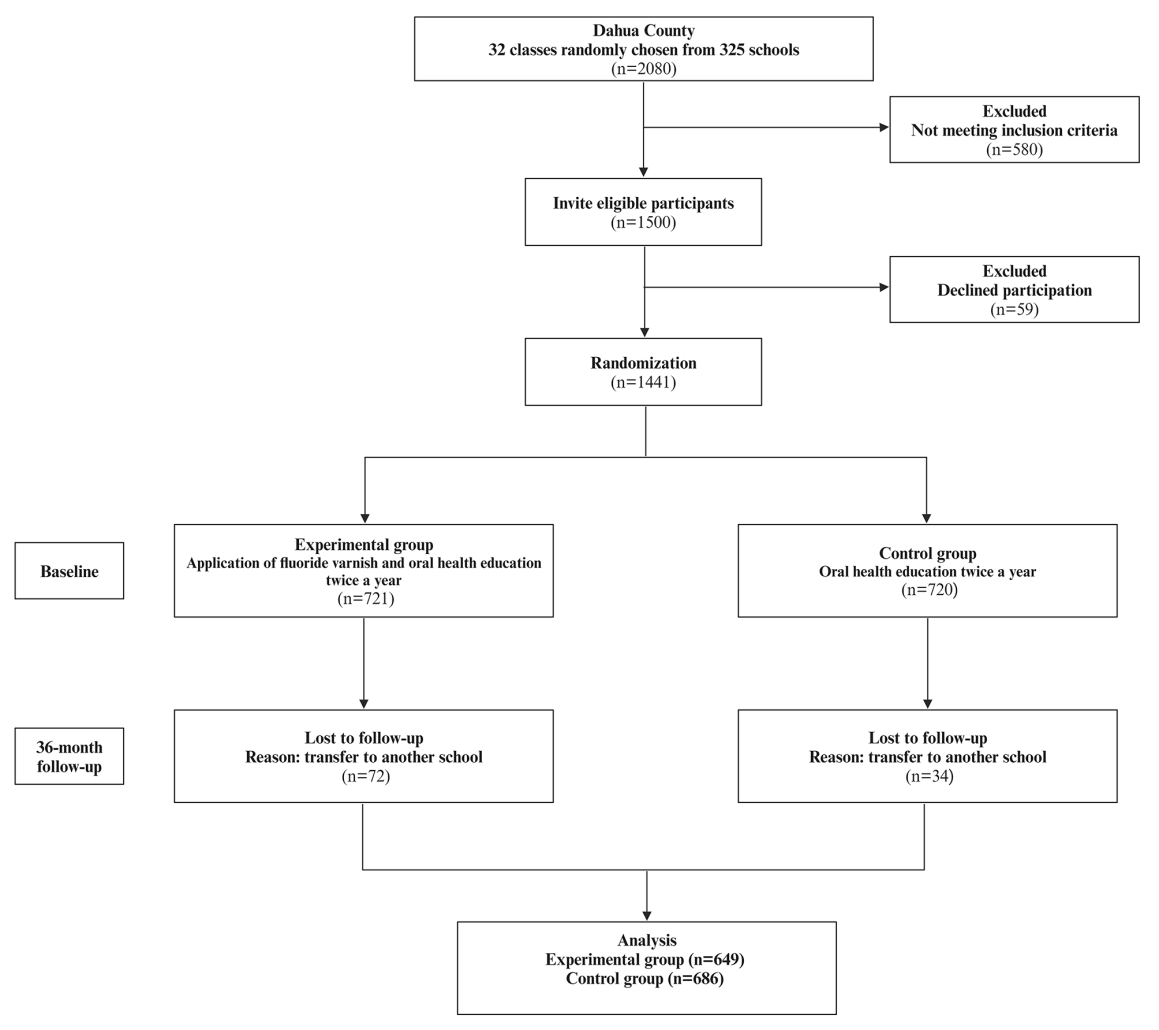
Study flowchart

Initially, 1,500 children were invited, but 59 declined, leaving 721 in the EG and 720 in the CG, for a 96.07% response rate. After 36 months, 106 children were lost to follow-up due to school transfers. Ultimately, a total of 1,335 children were included, with a second response rate of 92.64%. There were 649 children (376 boys, 273 girls) in the EG, with an average age of 6.96 ± 0.40 years. The CG included 686 children (383 boys, 303 girls), with an average age of 6.95 ± 0.40 years. There were no significant differences in sex or age between the two groups at baseline (*P* > 0.05) (Fig. 1).

After 3 years of intervention, all FPMs had erupted for the subjects. The prevalence of caries, DMFT index score and DMFS score of the FPMs in the EG were 50.85%, 1.12 and 1.58, respectively, and those in the CG were 59.04%, 1.36 and 1.93, respectively. The differences in the prevalence of caries, DMFT index score, and DMFS score of the FPMs were statistically significant between the two groups (*P* < 0.05; Table 1).

**Table 1:** Prevalence of dental caries, DMFT score, and DMFS score in the first permanent molars in the two groups of children after the intervention

Groups (N)	Prevalence*	X2	P	DMFT**	Z	P	DMFS**	Z	P
Experimental (649)	50.85(330)	9.041	0.003	1.12 ± 1.35	-3.273	0.001	1.58 ± 2.23	3.169	0.002
Control (686)	59.04(405)			1.36 ± 1.42			1.93 ± 2.40		

N, number of children. The data are presented as the % (n) and mean ± SD.

*Chi-square test; **Two independent samples t test

### Costs

Table 2 provides the related labour resource costs and consumable costs of the EG, with a total cost of 10,709.74 USD. For the CG, the total cost was 5,410.05 USD. The intervention cost per tooth was 4.13 USD in the EG and 1.97 USD in the CG.

**Table 2:** Details and costs of fluoride varnish applications

Cost category	Total at 36 months (USD)
**Cost of human resources**	
Dentist applying fluoride varnish	2,649.33
Dentist performing an oral exam	650.57
Oral health education	122.47
CDC staff	385.77
**Cost of materials**	
Fluoride varnish	2,914.73
Oral examination supplies	457.56
Toothpaste and toothbrushes	3,529.31
**Total costs**	10,709.74
Discount rate, %	3
Cost per child (*n* = 649)	16.50

The direct medical costs, transportation costs, and lost productivity costs were approximately 24.74 USD, 0.60 USD, and 18.69 USD, respectively, and the treatment cost of one carious tooth was 44.03 USD. Assuming that all caries were filled, the total cost, including filling costs, was 42,719.55 USD for the scheme with FV and 46,622.13 USD for the scheme without FV.

### Cost-effectiveness analysis

Compared with the CG, the EG had 209 fewer caries, with an ICER of 25.36 USD, that is, 25.36 USD per caries prevented by the application of FV compared with the control intervention (Table 3). Based on the treatment cost per caries, the WTP was estimated to be 44.03 USD.

**Table 3:** Cost effectiveness results for the two groups

Groups	Costs (USD)	Incremental costs (USD)	Caries lesions	Incremental caries	ICER
Experimental Control	10,709.74 5,410.05	5,299.69	727 936	209	25.36

### Cost–benefit analysis

Compared with the CG, the EG avoided 5,170.66 USD in medical expenses, 125.40 USD in transportation costs, and 3,906.21 USD in lost productivity costs. The CBR for the EG was 1.74 USD benefits per 1 USD cost (Table 4).

**Table 4:** The cost–benefit analysis of the two groups

Groups	Intervention costs (USD)	Incremental costs (USD)	Medical care costs averted (USD)	Transportation costs averted (USD)			Costs of lost productivity Averted (USD)		Cost–benefit ratio	
Experimental Control	10,709.74 5,410.05	5,299.69	5,170.66		125.40	3,906.21		1:1.74		

### Sensitivity analysis

**Fig. 2:**
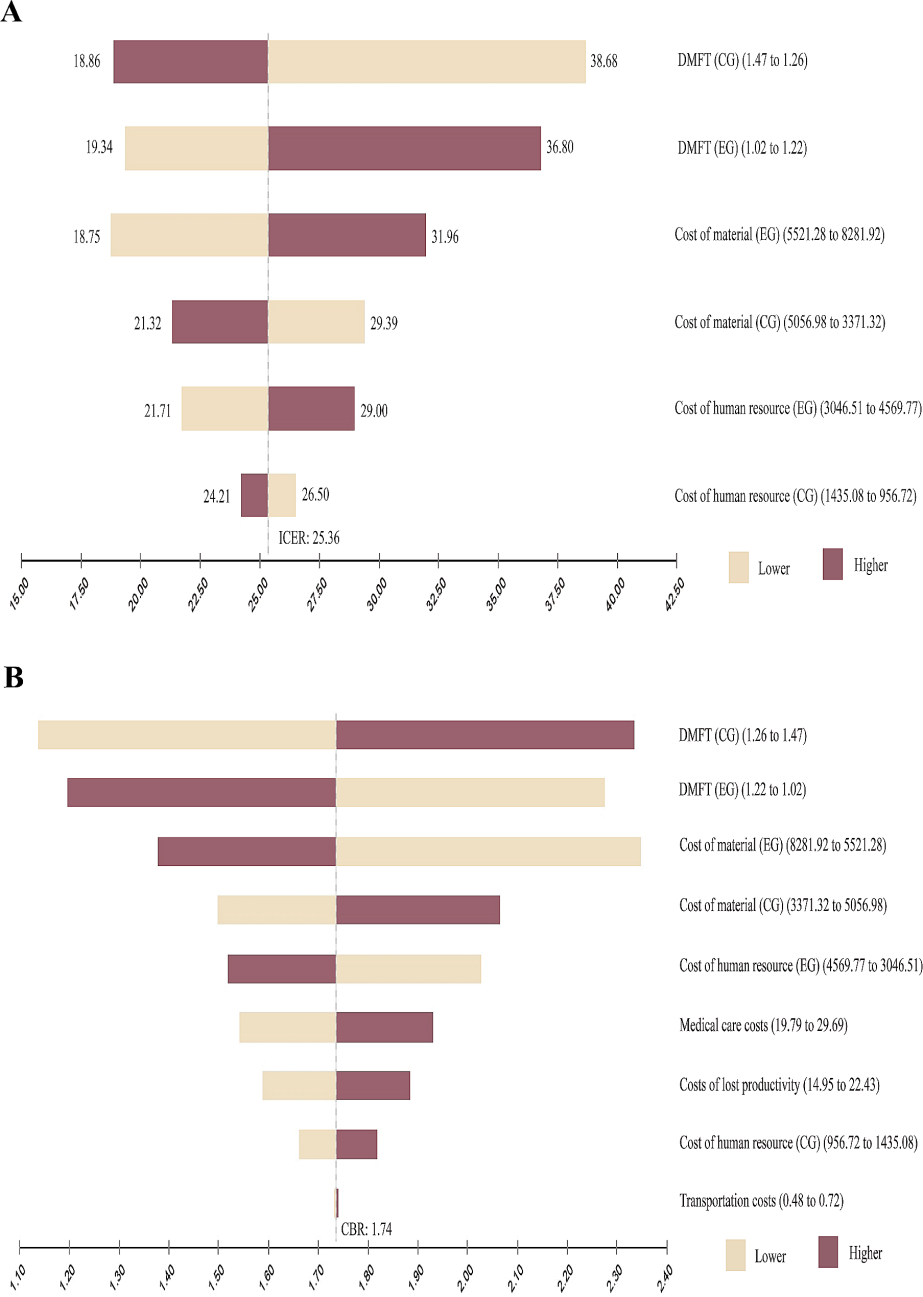
Tornado diagrams. (**A**) Change in the incremental cost-effectiveness ratio (ICER) in the case of unidirectional variation in each parameter. (**B**) The change in the cost–benefit ratio (CBR) in the case of unidirectional variation in each parameter. Yellow bars show the impact of an increase, and red bars show the impact of a decrease in the variable value

**Fig. 3:**
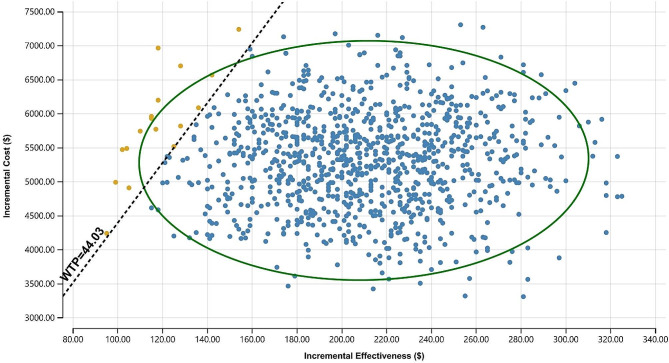
Incremental cost-effectiveness scatterplots of the EG versus the CG at a WTP threshold of 44.03 USD.

The parameters used to construct the base case are summarised in Tables 5 and 6. In Figs. 2 and 3, the tornado plots show how the ICER and CBR changed as the key variables changed. Through single-factor sensitivity analysis, the most critical factor affecting the ICER and CBR in this study was the average DMFT index score of the CG. The second key factor was the mean DMFT score of the EG. In Fig. 3, the probabilistic sensitivity analysis, presented as a scatter plot, shows that the probability of the ICER being lower than the WTP with a cost-effectiveness advantage was 98.3% at a WTP of 44.03 USD, and the model had good robustness.

**Table 5:** Overview of parameters that varied in the ICER-related sensitivity analysis

Parameters	Lower-case	Reference-case	Higher-case
Cost of human resources (EG)	-20%	100%	+ 20%
Cost of human resources (CG)	-20%	100%	+ 20%
Cost of materials (EG)	-20%	100%	+ 20%
Cost of materials (CG)	-20%	100%	+ 20%
DMFT, average score (EG)	1.02	1.12	1.22
DMFT, average score (CG)	1.26	1.36	1.47

**Table 6:** Overview of parameters that varied in the CBR-related sensitivity analysis

Parameters	Lower-case	Reference-case	Higher-case
Cost of human resources (EG)	-20%	100%	+ 20%
Cost of human resources (CG)	-20%	100%	+ 20%
Cost of materials (EG)	-20%	100%	+ 20%
Cost of materials (CG)	-20%	100%	+ 20%
Medical care costs	-20%	100%	+ 20%
Transportation costs	-20%	100%	+ 20%
Costs of lost productivity	-20%	100%	+ 20%
DMFT, average score (EG)	1.02	1.12	1.22
DMFT, average score (CG)	1.26	1.36	1.47

## Discussion

The effectiveness and economic analysis presented in our study underscore the significant potential of the use of FV combined with oral health education in rural settings. By focusing on the rural context of Guangxi, this study highlighted the unique challenges faced by these communities, including limited access to health care facilities, low levels of oral health awareness, and the prevalence of left-behind children. These factors increase the risk of dental caries, underscoring the urgency of implementing comprehensive, cost-effective preventive strategies [17].

During the 3-year period of FV application in this study, no adverse events were reported. A previous study showed that during a 10-year period of FV application in the United States, the estimated incidence rate of adverse events ranged from 0.099 to 0.105 per million applications [18]. The above findings indicate that FV can be considered a safe dental product. The high safety profile of FV is attributed to the small dosage required for application, its rapid curing and adherence to tooth surfaces, slow fluoride release, and a very low rate of fluorosis. In addition, FV has a relatively long lifespan and low technical sensitivity [19, 20]. Notably, in addition to FV, pit and fissure sealants are also considered effective preventive measures [9, 10]. A meta-analysis comparing the preventive effects of FV and resin-based fissure sealants on the FPMs of school-aged children revealed no significant difference in caries prevention between the two groups and further highlighted the affordability and ease of use of FV [21]. Therefore, given the safety, cost-effectiveness, and low technical sensitivity of FV application, FV is especially suitable for use and promotion in resource-limited rural areas.

The introduction of FV as a preventive measure is not only a clinical decision but also a public health strategy aimed at reducing the overall burden of dental diseases on the health care system. In the United States, the close relationship between dentists and community centres, as well as the strong cooperation and communication between doctors and policy supporters and the eligibility recognition of Medicaid issued by the government, effectively ensures the stable implementation of this recommended intervention [22]. However, a study from South Africa evaluated the effectiveness of FV in preventing caries among high-risk populations, revealing that, in addition to daily supervised tooth brushing, regular FV application had no significant effect on caries prevention [23]. This may be due to differences in national contexts and disparities between communities, as many countries and regions are still unable to effectively implement this approach [9, 24]. Therefore, based on this study, an integrated intervention strategy that combines oral health education with the application of FV is recommended. This approach aims to synergize the long-term benefits of educating individuals on proper oral hygiene practices with the immediate protective effects of fluoride varnish against dental caries.

Our study spanned three years, underscoring the critical role of time in the effectiveness of dental public health measures, particularly in the areas of oral health education and the application of FV. Sustained, long-term efforts in oral health education are essential for changing behaviours and enhancing knowledge. Additionally, FV must be reapplied at clinically recommended intervals to maintain its caries prevention benefits. Integrating these strategies effectively is crucial for maximising the effectiveness of interventions and ensuring the success of dental public health programs.

To the best of our knowledge, this is the first study to analyse the effectiveness of FV combined with oral health education in preventing caries in FPMs among children aged 6–8 years living in a remote rural area of China from a cost-effectiveness and cost-benefit perspective. Within the limitations of this experiment, we found that, after 36 months of intervention, the intervention cost of FV application plus oral health education was higher than those of oral health education alone, but the cost of the former was lower when the treatment cost of dental caries was considered. This was mainly because the intervention of FV application plus oral health education was more effective in preventing caries in FPMs, resulting in fewer caries, and the savings in caries treatment made up for the higher cost of the initial FV application. A study from Chile indicates that applying FV within a primary care setting is the most cost-effective strategy [25]. However, the prevalence of FPM caries among children in the intervention of FV application plus oral health education (50.85%) was still much greater than that in 7–9-year-old children in China (approximately 20%) [26]. This may be related to the fact that almost all left-behind children are cared for by elderly people who may not be educated enough to provide the children with oral knowledge and manage their oral behaviour. A study has shown that the prevalence of caries in the FPMs among second-grade students in Xiangyun, Yunnan, China, is 47.6%, with only 2.6% of the affected children receiving fillings. Our research may offer insights for regions experiencing similar situations [27].

We not only used a controlled design to evaluate effectiveness but also conducted an economic evaluation of the study by performing a CEA and a CBA [28]. For health care departments and public health decision-makers, a CEA can provide strong scientific evidence for the rational allocation of limited public health resources and the selection of the most effective health intervention [29, 30]. The ICER is highly relevant to decision-making regarding public health prevention programs. In this study, the intervention of FV application plus oral health education to prevent dental caries in children from remote areas was shown to be cost-effective and to reduce the caries burden, as the ICER was 25.36 USD, which was much lower than the WTP. In a sensitivity analysis, we found that the average DMFT index score was the variable with the greatest influence on the cost-effectiveness relationship, and the intervention of FV application plus oral health education was shown to be cost-effective even when the lower bound of the CI was considered. A study on rural children in non-fluoridated areas showed that the strategy of combining oral health education with FV application is more effective and cost-effective in preventing early childhood caries [31]. CBA involves selecting the optimal intervention program through comparison to obtain the maximum benefit with the minimum cost in health decision-making [32, 33]. In the present study, we found that the comprehensive intervention combining FV with oral health education for caries prevention was wholly beneficial. Even when the lower bound of the key parameters was considered, the CBR showed that the benefits value was still greater than one for every dollar invested in cost. Few existing studies have conducted CEA and CBA for caries interventions in children living in remote rural areas, and the differences in age of the target populations, outcome indicators, and national health care systems make comparisons with other studies difficult.

A limitation of this study was the lack of a blank control group, which could constrain the comprehensive assessment of the efficacy of the oral health education intervention. Future research should independently assess the effects of oral health education and FV application. Another limitation is that there was no placebo in the study design, and participants undergoing FV application may have been aware of their allocation, which could have resulted in placebo effects or influenced dropout rates. Furthermore, the differential loss to follow-up due to school transfers between the EG and CG (649 vs. 686 children) resulted in an imbalance in the final participant numbers, highlighting the inevitable issue of attrition in long-term studies. Additionally, engaging parents and caregivers in oral health education remains a significant challenge. Innovative methods leveraging technology and community networks may offer viable solutions to enhance engagement among this key demographic, especially in rural areas where traditional communication channels may be less effective.

## Conclusion

School-based combined interventions with FV application and oral health education have greater cost-effectiveness and cost-benefits than oral health education alone as public health interventions to prevent caries in the FPMs of children in poor rural areas. The evidence generated could support the implementation of such interventions as a viable strategy for improving oral health outcomes among children in rural, resource-constrained settings. The findings might be generalizable to similar parts of the world.

## Data Availability

The dates used and analysed in this study are available from the corresponding author upon reasonable request.

## Abbreviations

CBA: Cost–benefit analysis
CBR: Cost–benefit ratio
CDC: Centers for Disease Control
CEA: Cost-effectiveness analysis
CG: Control group
CHEERS: Consolidated Health Economic Evaluation Reporting Standards
DMFT: Decayed, Missing, and Filled Teeth
EG: Experimental group
FPM: First permanent molar
FV: Fluoride varnish
ICER: Incremental cost-effectiveness ratio
WHO: World Health Organization
WTP: Willingness to pay
